# 5-Methylcytosine and 5-Hydroxymethylcytosine Signatures Underlying Pediatric Cancers

**DOI:** 10.3390/epigenomes3020009

**Published:** 2019-05-09

**Authors:** Shalu Jhanwar, Ajinkya Deogade

**Affiliations:** 1 Developmental Genetics, Department of Biomedicine, University of Basel, 4058 Basel, Switzerland; 2 Centre for Genomic Regulation (CRG), The Barcelona Institute of Science and Technology, Universitat Pompeu Fabra (UPF), 08003 Barcelona, Spain; 3 Department of Molecular, Cellular and Developmental Biology, University of California, Santa Barbara, Santa Barbara, CA 93106, USA

**Keywords:** epigenetics, DNA methylation, pediatric cancer, 5mC, 5hmC, acute lymphoblastic leukaemia, neuroblastoma

## Abstract

In addition to the genetic variations, recent evidence has shown that DNA methylation of both 5-methylcytosine (5mC) and 5-hydroxymethylcytosine (5hmC) underlies the pathogenesis of pediatric cancer. Given the high mortality rate, there is an urgent need to study the mechanisms contributing to the pathogenicity of pediatric cancer. Over the past decades, next-generation sequencing (NGS) has enabled us to perform genome-wide screening to study the complex regulatory mechanisms of 5mC and 5hmC underlying pediatric tumorigenesis. To shed light on recent developments on pediatric cancer predisposition and tumor progression, here we discuss the role of both genome-wide and locus-specific dysregulation of 5mC and 5hmC in hematopoiesis malignancy and neuroblastoma, the most common types of pediatric cancer, together with their therapeutic potential.

## 1. Introduction

Biological instructions for a cell’s identity are encoded in its genetic makeup. In spite of sharing the same genome, the interpretation of these instructions varies among cell types. This is governed by epigenetics, which regulates the chromatin dynamics and gene expression. To primarily describe the mechanisms of cell fate and influence of the genetic processes on development, Conrad Waddington [1] coined the term *epigenesis* in 1942. Over the years, the purview of epigenesis has expanded and undergone an evolution. Modern epigenetics emphasizes the effect of changes in chromatin structure that are not encoded in the primary DNA sequence [2]. Epigenetics encompasses DNA methylation, histone modification, DNA accessibility, and chromatin structure, which regulate patterns of gene expression. The most common form of epigenetic modification is DNA methylation, which is brought about by covalent addition of the methyl group at the fifth carbon of the cytosine ring, resulting in 5-methylcytosine (5mC). DNA methyltransferases DNMT1, DNMT3A, and DNMT3B [3] catalyze the modification of cytosines to 5mC. Besides 5mC, the TET (ten-eleven translocation) family of dioxygenases execute the oxidation of 5mC, leading to the formation of 5-hydroxymethylcytosine (5hmC). Due to their ubiquitous presence and functional importance, 5mC and 5hmC are also considered the fifth and the sixth bases of the genome, respectively [4].

In recent years, epigenetic alterations, especially aberrant DNA methylation, are gaining recognition as primary drivers behind pediatric tumorigenesis [5]. Statistics from the year 2018 showed leukemia (29%) and nervous system tumors (26%) as the most prevalent types of pediatric cancers. Other cancer types, including lymphomas and reticuloendothelial neoplasms (12%), neuroblastoma (6%), and renal (Wilm’s) tumors (5%), are also found to be frequent among children [6]. In 2018, the United States had an overall pediatric cancer incidence of 10,500, with 11.24% ultimately resulting in death. Given the high mortality rate, there is an urgent need to study mechanisms contributing to the pathogenicity of pediatric cancer. Unlike adult malignancies, pediatric solid tumors harbor more epigenetic alterations relative to genetic mutations. In pediatric solid tumors, the epigenetic plasticity of the target cell drives the propagation of genetic lesions that cause initiation and maintenance of oncogenesis [7].

In past decades, next-generation sequencing (NGS) has been revolutionized the way we perform genome-wide screening to study complex regulatory mechanisms of 5mC and 5hmC underlying pediatric tumorigenesis [8]. Both loss (hypomethylation) and gain (hypermethylation) of DNA methylation are linked with cancer progression. It has been known for over three decades that cancer cells [9] and primary human tumors [10] have depleted levels of 5mC relative to normal tissues. Mechanistically, aberrant hypomethylation has been shown to contribute to tumorigenesis by (a) activating cancer-causing genes such as *c-MYC* [11] and *H-RAS* [10]; (b) by retrotransposon activation [12]; and (c) chromosome instability [13]. Similarly, growing evidence indicates tumor-associated loss of 5hmC, which is considered a crucial epigenetic hallmark of different cancers, including glioma [14], lung [15], cervical [16], breast [17], ovarian [18], and gastric [19] cancers.

In addition to the loss of methylation, the hypermethylation of CpG islands (CGI) in gene promoters is linked to tumorigenesis. Hypermethylation of CGI causes the repression of the tumor-suppressor gene expression [20], such as cell cycle regulators (P16^INK4a^, P15^INK4a^) and DNA repair genes (*BRCA1*). An elevated global level of 5mC and 5hmC has been reported in mixed-lineage leukemia (MLL), acute myeloid leukemia [21], and breast cancer [22]. Moreover, aberrations in cofactor biochemical pathways involving 2-oxoglutarate, together with mutations in *IDH1/2* [23], *SDH*, and *FH* [24] may alter levels of 5hmC and 5mC in certain types of tumors, in either a direct or indirect manner [25]. To shed light on recent developments on pediatric cancer predisposition and tumor progression, here we discuss the role of genome-wide and locus-specific dysregulation of 5mC and 5hmC in the most common type of pediatric cancer, i.e., hematopoiesis malignancy and neuroblastoma, together with their therapeutic potential.

## 2. 5mC and 5hmC Defects in Hematopoiesis Malignancies

Hematological malignancies arise due to either the dysfunction or misregulation of signal transduction pathways and molecular machinery, such as transcription factors and chromatin modifiers [26]. Acute lymphoblastic leukemia (ALL) is the most common childhood malignancy. High hyperdiploidy (HeH) and chromosomal translocations of *ETV6–RUNX1* are the two most frequent genetic alterations in childhood ALL, which account for 25% and 20% of pediatric ALL diagnoses, respectively [27]. Several independent studies have reported that aberrant promoter methylation, predominantly hypermethylated CGIs, are associated with a prognosis of ALL [28]. In particular, locus-specific aberrant promoter methylation of *PCDH17* [29] and *MGMT* [30] are considered to be a potential prognostic marker for pediatric ALL. DNA methylation arrays of pediatric ALL patients revealed aberrant 5mC levels in patients with chromosomal translocations of *ETV6–RUNX1* [31]. Additionally, hypomethylation hotspots and lower 5mC levels of aneuploid chromosomes were found to be the most striking epigenetic features of HeH [32,33]. Interestingly, an integrated methylome and transcriptome analysis comprising 19 pre-B pediatric ALL patient samples revealed epi-driver genes such as *SYNE1*, *PAWR*, and *HDAC9* [34]. Moreover, the study found potential enhancers within intronic and intergenic differentially methylated regions (DMR), called enhancer DMR (eDMR). These findings corroborate that altered enhancer methylation may contribute to the pathogenesis of pediatric ALL. An interesting study showed reduced levels of 5hmC in one percent of ALL cases due to mutations in the enzyme *TET2* [35].

Overall, the aforementioned studies highlight the potential role of 5mC and 5hmC as key drivers of pediatric leukemogenesis. However, the molecular basis for hematological alterations by these epigenetic modifiers still remains elusive. Specifically, how do aberrations of 5mC and 5hmC cause transcriptional misregulation of target genes is not fully understood. Perhaps, identifying new therapeutic target genes specific to different ALL subtypes and perturbation of those genes either alone or in combination may allow us to determine the molecular basis of these alterations during hematopoietic transformation.

## 3. 5mC and 5hmC Defects in Neuroblastoma

Neuroblastoma (NB) is an extracranial solid tumor that occurs due to improper maturation of the progenitor cells from the sympathoadrenal lineage [36]. NB is developed at an early age in children and has a high frequency of metastasis at diagnosis [37]. Based on its anatomical presence at diagnosis and imaging, two different tumor classification systems have been proposed for pediatric NB: a) Stage 1, 2A, 2B, 3, 4, and 4S by the International Neuroblastoma Staging System Committee (INSS); and b) Stage L1, L2, M, and MS by The International Neuroblastoma Risk Group Staging System (INRGSS).

Recent studies have reported the contribution of both hypo- and hypermethylation of 5mC and 5hmC in pediatric NB. Genome-wide methylation profiling of tumors revealed hypermethylation of CpGs and non-CpGs in M and L stages of NB respectively [38]. In particular, sets of genes with hypermethylated CpG sites include a) *TERT*, *PCDHGA4*, *DLX5*, and *DLX6-AS1* in M tumors [39]; and b) *ANXA11*, *MAT1A*, *PIK3AP1*, *RLBP1* and *SNED1* in high-risk tumors [40]. Interestingly, Stage 4S NB tumors show characteristic hypermethylation of specific subtelomeric promoters [41]. Genome-wide comparison of methylation levels between NB and the neural crest precursor cells found *MEGF10* to be epigenetically silenced by DNA hypermethylation in NB cell lines [39]. Furthermore, the knock-down of *MEGF10* expression in NB cell lines promoted cell growth, highlighting the clinical significance of the methylation of tumor-suppressing genes [39]. An interesting study by Mariani et al. found a significant rise in 5hmC levels in the regulatory regions of NB-associated genes such as *ENO1*, *PGK1*, *SLCO4A1*, *HK2*, and *HILPDA* in hypoxic conditions [42]. In addition to hypermethylation, a genome-wide comparison of 35 primary NB tumors with human embryonic stem cells (hESC) found hypomethylation—predominantly outside of CGIs and associated with quiescent and polycomb repressed domains [40].

Taken together, the above studies have identified specific aberrant DNA methylation signatures contributing to different stages of NB. However, the current risk classification system poorly predicts high-risk patients that are more likely to respond to treatments. To refine the current risk stratification and development of novel therapeutics, future studies should emphasize (a) exploring the 5mC and 5hmC mediated molecular cues underlying the growth and survival of high-risk pediatric NB; and (b) an in-depth epigenetic and transcriptional analysis of distinct neuroblastoma samples.

## 4. 5mc and 5hmC Technological Advancements

Several assays are available for the discovery and validation of 5mC and 5hmC associated biomarkers. The experimental methods for the interrogation of 5mC can be broadly categorized into (a) genomic DNA digestion with methyl-sensitive restriction enzymes (MRE-seq); (b) affinity-based enrichment of methylated DNA regions (MeDIP-seq); (c) use of chemical conversion methods—bisulfite treatment (RRBS, WGBS) [43]. However, these approaches are unable to discriminate between 5hmC and 5mC. Fortunately, past decades have seen rapid technological advancement that has given rise to diverse approaches for the systematic detection of 5hmC, including (a) whole-genome bisulfite/oxidative bisulfite sequencing (WG Bis/OxBis-seq); (b) Infinium HumanMethylation450 BeadChip arrays coupled with oxidative bisulfite sequencing (HM450K Bis/OxBis); and (c) antibody-based immunoprecipitation and sequencing of hydroxymethylated DNA (hMeDIP-seq) [44]. Additionally, locus specific characterization of 5mC and 5hmC using TAB-seq is also feasible and highly useful for the validation of candidate regions [44]. 

The existing experimental procedures for the detection of 5mC and 5hmC vary with respect to the cost, coverage, quantitative accuracy, and efficiency. An informed choice of the best-suited method can be made based on the aims and specific requirements of the study, amount and quality of the DNA sample(s), and sequencing cost. Motivated by the ever-increasing wealth and diversity of large-scale DNA methylation datasets, a variety of computational approaches have been developed to aid analysis of 5mC and 5hmC NGS datasets. A comprehensive list of tools and a workflow summarizing all the necessary steps required for 5mC analysis is provided in [43].

## 5. Conclusions

In this editorial, we have outlined the critical contribution of 5mC and 5hmC dysregulation as a driving force behind the origin and progression of pediatric tumors, in particular to pediatric ALL and NB. Given a very stable molecular memory of the progenitor cell and tumor subtype-specific signatures, 5mC and 5hmC can act as potential biomarkers to characterize lineage, improve risk stratification, and develop epigenetically targeted therapies for pediatric malignancies. A major challenge is the absence or minimal overlap of identified markers across different studies [45]. Perhaps the continually growing experimental data representing distinct tumor subgroups and the usage of standard analytical approaches can minimize such variability. Moreover, future analysis of DNA methylation at the single-cell stage would be extremely useful to understand complex heterogeneous tumor populations and may further improve future clinical medication.

## Abbreviations

NGS	Next generation Sequencing
hESC	Human embryonic stem cells
RRBS	Reduced representation bisulfite sequencing
WGBS	Whole-Genome Bisulfite Sequencing
5mC	5-Methylcytosine
5hmC	5-Hydroxymethylcytosine
ALL	Acute lymphoblastic leukaemia
NB	Neuroblastoma
IDH1/2	Isocitrate dehydrogenase 1 and 2
SDH	Succinate dehydrogenase
FH	Fumarate hydratase
TET2	Ten-Eleven-Translocation 2
